# Survey of Mechanical Properties of Geopolymer Concrete: A Comprehensive Review and Data Analysis

**DOI:** 10.3390/ma14164690

**Published:** 2021-08-20

**Authors:** Azad A. Mohammed, Hemn Unis Ahmed, Amir Mosavi

**Affiliations:** 1 Civil Engineering Department, College of Engineering, University of Sulaimani, Kurdistan Region, Sulaimaniyah 46001, Iraq; azad.mohammed@univsul.edu.iq; 2 John von Neumann Faculty of Informatics, Obuda University, 1034 Budapest, Hungary

**Keywords:** geopolymer concrete, mechanical properties, data analysis, compressive strength, flexural strength, splitting tensile strength, elastic modulus, review, state of the art, survey

## Abstract

Mechanical properties and data analysis for the prediction of different mechanical properties of geopolymer concrete (GPC) were investigated. A relatively large amount of test data from 126 past works was collected, analyzed, and correlation between different mechanical properties and compressive strength was investigated. Equations were proposed for the properties of splitting tensile strength, flexural strength, modulus of elasticity, Poisson’s ratio, and strain corresponding to peak compressive strength. The proposed equations were found accurate and can be used to prepare a state-of-art report on GPC. Based on data analysis, it was found that there is a chance to apply some past proposed equations for predicting different mechanical properties. CEB-FIP equations for the prediction of splitting tensile strength and strain corresponding to peak compressive stress were found to be accurate, while ACI 318 equations for splitting tensile and elastic modulus overestimates test data for GPC of low compressive strength.

## 1. Introduction

Initial research on geopolymers carried out by Davidovits [1] was on the linear organic polymer, which is a branch of organic chemistry. Later, this topic was extended beyond this scope and research conducted in the early 1970s was focused on developing nonflammable inorganic polymer materials suitable for fire resistance. This attempt was because of the fact that the used organic polymers at that time were of low heat resistance. The work was ended to develop an amorphous to semi-crystalline three-dimensional silico-aluminate composite called geopolymer. This invention was followed by the manufacturing of fire-resistant chipboard panels, different geopolymeric ceramics, and later, geopolymeric binders including high strength cement and fireproof geopolymer fiber reinforced composites [1].

Geopolymer is essentially different from the conventional concrete which consists of hydraulic cement as a binder. Instead, there is an alkali-activated mineral admixture as a binding medium holding an inert aggregate to form a compact mass. This new type of concrete can offer many benefits including high early strength [2], high temperature resistance [3], and good chemical resistance for aggressive environments [4], as compared with normal concrete. In particular, durability of ordinary Portland cement concrete is under examination, as many concrete structures, especially those built in corrosive environments, start to deteriorate after 20 to 30 years [5]. Other reasons for using geopolymer concrete (GPC) include the vital need to save the natural environment and to use cleaner construction material since there is a global warning against the use of Portland cement-based composite. It is clear that the production of Portland cement accompanies the use of natural resources including gravel, water, and raw materials required for manufacturing of cement, leading to destroying the surrounding environment. Reports showed that about 2.7 billion tonnes of the raw materials needed every year for cement manufacturing [6]. Other reports [7] indicate that in order to manufacture one ton of Portland cement there is a need for about 2.8 tons of raw materials, including fuel. Another problem to be considered is the environmental pollution encountered with production of Portland cement. During the manufacturing of Portland cement, large amounts of greenhouse gas (CO_2_) will be released into the atmosphere, and the related reports indicate that the cement industry contributes around 8% of the worldwide yearly CO_2_ emission [8].

Based on the above-mentioned facts, the problem-related use of concrete must be well addressed, and there is a vital need to reduce Portland cement concrete consuming or searching for the alternatives for construction purposes. In a recent paper [9], the performance of composite concrete-timber section for roof construction was investigated, and the authors concluded that there is a chance to reduce concrete thickness by one half if populous nigari joist is used to make a composite section. Geopolymers seem to be a good solution to produce a clean concrete, since the Portland cement can be totally replaced, and instead there is a special concrete depending on an alkali-activated pozzolanic material, such as fly ash and blast furnace slag, to provide a binding medium. Physical and mechanical properties of fly ash (FA)-based geopolymer concrete (GPC), compared to those of Portland cement concrete (PCC), were investigated by Nikoloutsopoulos et al. [10] through testing three GPCs with different FA content and three appropriate PCC. It was shown that in some cases, minor adjustments of the regulations are needed, while in other cases complete revision is required. GPC indicated competitive compressive strength compared to PCC, while modulus of elasticity was about 50% less than that of PCC. GPC shows a higher mid-span deflection during flexural test up to 35% compared with that of PCC. Furthermore, ultrasonic pulse velocity of GPC was found quite different from that of PCC, even for the same strength level. They concluded that the quality of GPC cannot be assessed using the classification table used for PCC. The ratio of binder (FA) to aggregates seems to have a significant effect on the properties of GPC, in which GPC with 750 kg/m^3^ FA seems to be the best choice with regard engineering and environmental criteria.

It was reported that the frost resistance of alkali-activated materials (AAM) is very good [11]. This was confirmed by investigating mechanical properties of GPC and frost resistance of different compositions of alkali activators made of sodium water glass with a silicate modulus modified with potassium hydroxide. Bilek et al. [11] found that the strengths of AAMs are significantly affected by the curing method, while the frost resistance depends on the method of curing and on the composition of the activator. As a conclusion, good frost resistance can be achieved if: (a) the optimal ratio between the alkalis and silica in the activator, in which activation with hydroxide or with the water glass with a high silicate modulus (low (Na_2_O + K_2_O)/SiO_2_ (R/S) mass ratios, was found not suitable. The optimal R/S was recommended to be between 50/50 to 70/30; (b) the optimal amount of activator—dry mass of the activator higher than 15% seems to be deleterious from the point of view of frost resistance, knowing that the strengths of these materials are very high.

Researchers are now investigating the performance of geopolymer concrete for manufacturing railway sleepers where the sleepers are subjected to millions of cyclic loads. The railway sleeper should be electrically nonconductive and geopolymer concrete seems to be excellent for this purpose because it has superior electrical resistance [12]. The structural performance of geopolymer concrete filled hybrid composite beams was investigated by Ferdous et al. [12]. Three hybrid beams filled with geopolymer concrete were prepared and tested to evaluate their flexural behavior. A numerical and analytical evaluation of the behavior of hybrid beam was performed and results showed a good agreement with the experimental investigation. Furthermore, the suitability of the beam for a composite railway sleeper was evaluated and compared with existing timber and composite sleepers. Additionally, the beams’ performance in a ballast railway track was analyzed using Strand7 finite element simulation software. The new concept of using geopolymer concrete as infill to pultrude composite sections satisfied the stiffness and strength requirements for a railway sleeper.

Different properties of geopolymer concrete were extensively studied in the past 20 years, and there is a relatively large amount of experimental test data. Additionally, there are many proposed equations to calculate different mechanical properties. The main goal of the current study is to perform a revised data analysis for the mechanical properties of geopolymer concrete which was not carried out in the literature. First, a state-of-art review was made for different mechanical properties of GPC followed by a revised data analysis among the mechanical properties of GPC including compressive strength, splitting tensile strength, flexural strength, modulus of elasticity, Poisson’s ratio and strain corresponding to peak compressive stress. Data analysis based on a large number of experimental observations is of great importance to develop equations for calculating different concrete properties for global applications. Because, the proposed equations developed by some researchers are based on tests performed on local materials or based on a few research works. The proposed equations obtained in this study are based on a relatively large amount of test data accompanied with accuracy since they based on different data sources. The proposed equations for different mechanical properties can be used to prepare a state-of-art report on GPC properties and could be used for the design GPC structural members.

## 2. State-of-the-Art Review of Mechanical Properties of Geopolymer Concrete

Understanding mechanical properties of GPC is an important step toward producing large quantities of GPC with reasonably consistent and predictive engineering properties. These properties were the subject of numerous investigations in the past 20 years. The authors have been reviewed more than 250 research works on this topic and found that there are many ways to produce the geopolymers of different properties. Below, reviewing of important mechanical properties have been done and those parameters governing each property and relation among them are briefly investigated. Important parameters governing the performance of geopolymer binder are (a) activator solution-to-source material (fly ash, slag, etc.) ratio, (b) concentration of NaOH solution (molarity), (c) sodium silicate solution-to-sodium hydroxide solution ratio (Na_2_SiO_3_/NaOH), and this parameter depends on the composition of the sodium silicate solution, (d) curing temperature, (e) curing period, and (f) water content [10]. Indeed, if the basic pozzolanic material is partially replaced with other materials, there is a chance to adjust the binding characteristics

Different properties of geopolymer paste [13,14], mortar [14], and concrete [15] were experimentally investigated. If the density of concrete is considered, there are two types of geopolymer concrete, normal weight and lightweight, and the latter may be foamed concrete [16], or others based on lightweight aggregate [17,18]. Properties of self-compacting geopolymer concrete were experimentally investigated by Memon et al. [19], Ushaa et al. [20] and Saini and Vattifalli [21]. Behavior of GPC with nanomaterials was investigated by Phoo-ngernkham et al. [14]. Pozzolanic materials used for GPC mixes were mostly class F fly ash; however, class C fly ash [22], Phoo-ngernkham et al. [23], natural Pozzolan [24], ground granulated blast furnace slag (GGBS) [25,26], metakaolin [27,28], rice husk ash [29], a mixture of two or more Pozzolanic materials [30,31,32], and ceramic dust waste [33] were also examined. Some special ashes or compounds were used by some investigators such as palm oil fuel ash (POFA) [34], waste bottle glass (WBG) [35], and sugarcane bagasse ash (SCBA) [36,37]. With regard to the curing of GPC, several methods of curing were attempted by the researchers, including oven heating, membrane curing, steam curing, hot gunny curing, hydrothermal curing, room temperature, and water curing. Among them, oven curing proved to be the most efficient [38]. Heat curing regime of GPC depending on both temperature and duration, and initial temperature for curing varied between 30 °C [38] and 120 °C [39] or normally cured at the ambient temperature [23,25], while curing time up to 110 h was attempted [40]. Below, important mechanical properties of GPC are mentioned and discussed.

### 2.1. Compressive Strength

This property was extensively investigated in the laboratory and majority of research works on geopolymer concrete contained data on this property. Those parameters governing compressive strength of GPC are briefly discussed herein. Shehab et al. [41] observed that the values of compressive strength, bond strength, splitting tensile strength and flexural strength are the highest at 50% ordinary Portland cement (OPC) replacement with fly ash, while Vijai et al. [42] found that replacement of 10% of fly ash by OPC in GPC mix resulted in an enhanced compressive strength, split tensile strength and flexural strength. Tests by Lloyd and Rangan [43] showed that the inclusion of a 24 h period before curing increased the compressive strength of GPC. Curing at ambient condition will produce low early strength concrete, while there is a significant strength improvement on using high temperature. It should be noted that extended curing time able to enhance the geopolymerization mechanism and consequently the strength; however, longer duration of curing at an elevated temperature results in failure of the concrete [44]. In general, higher initial curing temperature and duration resulted in higher compressive strength [15,45,46,47]. Experimental tests by Adam and Horianto [39] showed that both temperature and duration of initial heat curing plays a major role for the strength development of fly ash-based geopolymer mortar. The optimum heat curing regime was found to be at 120° for 20 h. Tests by Joseph and Mathew [48] indicate 100 °C as the best temperature, while the optimum time of curing at 60 °C observed by Chindaprasirt et al. [49] was 3 h. These researchers found that the optimum curing temperature is 75 °C. The reaction was completed at 7 days to obtain the maximum strength and no further strength was observed. The importance of initial heat curing was also observed by Vijai et al. [42], Abdullah et al. [50] and Almuhsin et al. [51]. The latter researchers found an increase of 56% in the compressive strength for concrete subjected to one hour of oven curing at 90 °C. Increasing heat curing time to 90 h [52,53], and 110 h [40,54] resulted in an increase in compressive strength. Duration of heat curing was also investigated by Görhan and Kürklü [55], in which they found that there is an increase in compressive strength when heat curing (65 °C and 85 °C) increased from 5 to 24 h. Curing time more than 24 h was found has no appreciable effect on the strength [48].

Tests by Sathish Kumar et al. [56] indicate that the ratio of 7 days to 28 days compressive strength of ternary blend GPC is between 88% and 90%. Other tests by Nguyen et al. [2] showed that more than 93% of the 28-day compressive strength can be achieved at 7 days, regardless of fly ash type, heat curing method, or fly ash (FA) replacement with GGBS. In contrast, tests by Chi [57] indicate that the ratio is 88% for mortar cured at 65 °C which is larger than 66% when normally cured. For the metakaolin-based GPC mix subjected to normal air curing, the ratio of 7 days to 28 days compressive strength was found to be 73% and 88% [28]. Tests on self-compacting GPC based on fly ash and metakaolin show that the 7 days compressive strength value is close to the 28 days strength [58].

According to Nguyen et al. [2], increasing water/solid ratio from 0.2 to 0.3, can decrease the compressive strength of the FA-based GPC for alkaline-to-binder ratios of 0.3 and 0.4, while tests by Ahmad [59] for GPC subjected to initial curing at 70 °C (Oven) for 24 h showed that the optimum water/binder ratio is 0.25 to obtain maximum compressive strength. There was a strength increase with increasing alkali/fly ash ratio up to 0.45, lower than 0.5 measured by Al Bakri et al. [60] and Abdullah et al. [50]. With regard the liquid alkali/fly ash ratio the optimum value was observed to be 0.4 [61,62], while tests by Phoo-ngernkham and Phiangphimai [23] indicate a compressive strength reduction with increasing alkali activator solution/fly ash ratio from 0.4 to 0.9 for both M10 and M15 NaOH solutions. For GPC based on GGBS, optimal composition of solid/liquid ratio was noted to be 3.0 indicating the ratio of 0.33 for the alkali/GGBS ratio.

Al Bakri et al. [60] tested GPC of initial curing at 70 °C (Oven) for 24 h, the maximum compressive strength was for the mix of Na_2_SiO_3_/NaOH equal to 2.5. The same observation was made by Abdullah et al. [50], Aliabdo et al. [62], Aziz et al. [26], Joseph and Mathew [48], and Hadi et al. [63]. However, Vora and Dave [52] reported that the ratio of 2 resulted into a higher compressive strength. A value of 1.17 was found the best for GPC under ambient curing [51]. Other tests by Niş [64] showed that the critical silicate modulus depends on the molarity, in which for 14 M there was a ratio of 1, while for lower molarities the recommended value is 2. This finding supports that obtained previously by Rattanasak and Chindaprasirt [65]. Furthermore, a value of 1.5 was found to be the optimum for 10 M according to tests by Sathonsaowaphak et al. [61]. The use of a mix of NaOH and sodium silicate with a ratio of 1:1(SiO_2_/Na_2_O = 8) was able to activate the geopolymerization of fly ash [66].

NaOH solution molarity value for the alkali solution was found to be 12 for the highest compressive strength [50,59,60], while other tests [20,52,56,63,67] showed that the best molarity is 14. These experiments are not compatible with that performed by Raijiwala and Patil [68], Aliabdo et al. [62], Rachmansyah et al. [69] and Mathew and Issac [70] in which the molarity of 16 gives the highest compressive strength. In contrast, tests by Samantasinghar and Singh [71] indicate molarity of 8 for the maximum compressive strength.

Experiments by Puertas et al. [72] and Rajini et al. [73] showed that maximum compressive strength is related to 100% GGBS regardless the curing condition and any replacement of slag with fly ash resulted in the strength loss. Similarly, Guru Jawahar and Mounika [74] and Hadi et al. [63] found that the maximum compressive strength is related to the use of GGBS and if this material is partially replaced with fly ash or silica fume or metakaolin there is a strength loss, or there is a strength enhancement when the basic Pozzolana is replaced with GGBS [75]. The latter authors concluded that the replacement of fly ash with GGBS is a suitable alternative to oven curing. Nearly the same results were obtained by Bhargav and Kumar [76], Sarvanan and Elavanil [77] and Chidhambar and Manjunath [37]. The superiority of slag on fly ash for GPC subjected to different curing regimes was also observed by Kurtoğlu et al. [78]. Other tests showed that replacement of fly ash by GGBS up to 30% leads to an increase in compressive strength regardless of the curing temperature [2]. On the other hand, Yunsheng et al. [27] and Abhilash et al. [79] reported that the maximum compressive strength is related to replacing 50% metakaolin with GGBS. The same observation was also made by Raut et al. [80], Mathew and Issac [70] and Navyashree and Mogaveera [81] on geopolymer concrete made of fly ash replaced by GGBS. According to Okoya et al. [82], replacement of fly ash with silica fume up to 40% was found to be helpful to enhance compressive strength.

Using superplasticizers had very little effect on the compressive strength up to about 2% of this admixture to the amount of fly ash by mass [15]. This finding was supported by the observations of Malkawi et al. [83]. A reduction of compressive strength of GPC was observed when the superplasticizer dosage increased from 2% to 4% [52], while no significant change of compressive strength was observed by Aliabdo et al. [62] with increasing superplasticizer content.

It is of interest to mention the effect of other parameters influencing the strength of GPC. Tests by Joseph and Mathew [48] showed that the best total aggregate is 70% and the ratio of fine/coarse aggregate is 35% for mix of alkali/fly ash of 0.55. Saini and Vattifalli [21] found that addition of 2% nano silica resulted in improved workability, mechanical and durability performance of self-compacting GPC. Tests by Savitha et al. [36] showed that 5% replacement of GGBS by sugarcane bagasse ash (SCBA) gives highest compressive strength. On the other hand, compressive strength, splitting tensile strength, and elastic modulus of fly ash GPC improved with the increase of calcium aluminate cement [84].

### 2.2. Splitting Tensile Strength and Flexural Strength

Indirect tensile strength and flexural strength following similar trend of compressive strength of GPC [31,68], and in general, increasing compressive strength is accompanying with both splitting tensile (*f_sp_*) and flexural strengths (*f_r_*) enhancement. Consequently, those parameters governing compressive strength discussed in Section 2.1 govern these two properties. Test results by Hardijito [15] showed that the splitting tensile strength of geopolymer concrete is only a fraction of the compressive strength. However, there are some deviations from this general response described by some investigators.

Ryu et al. [66] reported that the rate of tensile strength increase slows with an increase of the compressive strength. Replacing fly ash with GGBS was found to have lower effect on splitting tensile and flexural strengths as compared with that on compressive strength [79]. Tests by Oderji et al. [85] showed a reduction in flexural strength as the fly ash replacement with slag increased from 15% to 20%, knowing that there is a compressive strength enhancement with this modification. Test data by Hassan et al. [46] showed that in contrast to elastic modulus of GPC the compressive and flexural strengths are enhanced well as a result of preheating of concrete at 75 °C for 26 h. Other tests by Sarvanan and Elavenil [77] showed that in contrast to the compressive strength, if 50% of fly ash is replaced with GGBS, there is a significant splitting tensile strength enhancement. The same observation was made for the elastic modulus property. Comparing data given by Partha et al. [30] with the others showed that using a special heat curing has an effect to enhance the flexure/compression ratio and to a lesser degree the tensile/compression ratio, as compared with the case of ambient temperature curing.

### 2.3. Modulus of Elasticity and Poisson’s Ratio

Modulus of elasticity (*E_c_*) follows similar trend of compressive strength of GPC, and according to tests by Hardijito [15], modulus of elasticity of GPC is increased with increasing compressive strength. Nath and Sarker [86] found that curing regime has no appreciable effect on the elastic modulus of GPC. Tests by Sarvanan and Elavenil [77] showed that in contrast to the compressive strength, if 50% of fly ash is replaced with GGBS, there is a significant elastic modulus enhancement.

With regard the Poisson’s ratio, this property has not been investigated as well compared with the other properties and consequently there is limited test data. The values of Poisson’s ratio fall between 0.23 and 0.26, which is slightly higher than the values assigned for normal strength OPC-based concrete [38]. Lower values of Poisson’s ratio were observed by other researchers for different GPCs [16,17,87,88]. In contrast to the other mentioned properties, there is no regular change with the compressive strength of GPC. This property tends to reduce with compressive strength reduction [16], while Sofi et al. [38] found an increase of Poisson’s ratio with increasing compressive strength. Other tests show that in contrast to other properties of GPC, increasing fly ash replacement with GGBS will lead to reducing the Poisson’s ratio [89]. In total, this behavior may complicate the work on developing equations for predicting Poisson’s ratio; however, this problem has been solved in the current investigation.

## 3. Proposed Equations for Mechanical Properties of GPC

Average direct tensile strength was found to be 0.12 times the compressive strength according to tests by Yellaiah et al. [90] on geopolymer mortar. The ratio of splitting tensile strength is 13.3% according to test Shehab et al. [41]. According to Ryu et al. [66], splitting tensile strength runs approximately from 7.8 to 8.2%, and this ratio is similar to that of normal concrete. They proposed a power equation for the tensile strength based on their own data. Review of literature revealed that with increasing compressive strength, the rate of increase of the tensile strength decreased [47]. Cui et al. [91] proposed a linear equation representing splitting tensile strength- compressive strength relationship GPC from regression analysis on 40 pairs of data sets from six published works. Based on 41 pair of data values on GPC of varying curing regimes, Llyoid and Rangan [43] have found that *f_sp_* is a function of square root of *f_c_’*. According to Vijai et al. [42] and Gomma et al. [92] *f_sp_* is a function of √*f_c_’*, while Jindal et al. [47] and Topark-Ngarm et al. [22] have found that the power equation is accurate. Compared to their tests data, Kurtoğlu et al. [78] reported that the equations of ACI 363 [93] and CEB-FIP model [94] for *f_sp_* can be applicable for structural design of GPC.

Yellaiah et al. [90] reported that average *f_r_* is 0.18 times the compressive strength, while Shehab et al. [41] found that the ratio is 10.98%. According to Hassan et al. [46], *f_r_* is a function of √*f_c_’*, but two different equations were proposed for the heat-cured and ambient-cured GPC. Additionally, Vijai et al. [42] found the same correlation between the two strengths, while Jindal et al. [47] obtained an equation similar to that of ACI 318 [95]. Based on their own test data, Phoo-ngernkham et al. [14] and Nath and Sarker [86] found that *f_r_* is a function of √*f_c_’*. Nath and Sarker [86] concluded that the equation recommended by AS 3600 [96] can be used for conservative prediction of *f_r_* of ambient-cured GPC.

The equations provided by AS 3600 [93], ACI 318 [95], and CEB-FIP [94] model code were found to overestimate the value of elastic modulus for GPC, and for this purpose Nath and Sarker [86] proposed an equation to predict the modulus of elasticity of GPC cured in ambient condition. According to Wongpa et al. [97], Phoo-ngernkham et al. [13], Nath and Sarker [86] and Hassan et al. [46]. *E_c_* is a function of √*f_c_’*. Cui et al. [91] proposed a power equation for the relationship between elastic modulus and compressive strength based on 35 pairs of data sets from four published works.

As compressive stress-strain relationship concerns, review of literature indicates that no attempt was made to develop models for the prediction based on based on regression analysis. Instead, the research were attempted to check the applicability of the current proposals derived for normal concrete on GPC. Hardjito [15] found that the stress-strain relations of fly ash-based GPC can be predicted using the equations developed for Portland cement concrete by Collins et al. [98]. Albitar et al. [32] found that the expressions of Hognestad [99] and Collins et al. [98] provide reasonable accuracy for slag GPC stress-strain relationships. Furthermore, Sarkar [100] used the stress-strain relationship by Popovics [101] modified by Thorenfeldt et al. [102] in the analysis of GPC columns.

## 4. Methodology and Materials

As one can find from literature review, the different proposed equations for the mechanical properties were based on the researcher’s own experiment or based on limited number of test data taken from several experimental studies. Since there is a large number of test data and the source of materials and test conditions are different from each other, this fact limits the applicability of the available equations, or at least must be checked based on a large number of test observations. For this purpose, there is a need for refined data analysis and developing new equations for the mechanical properties of GPC based on a global test data. Ryu et al. [66] reported that more reliable relationship between the compressive strength and the splitting tensile strength of GPC could be proposed after gathering a larger amount of data. In this study, an extensive search was made to collect a relatively large amount of test data for the properties of compressive strength, splitting tensile strength, flexural strength, elastic modulus, Poisson’s ratio, and strain corresponding to peak compressive stress.

A total of 1358 dataset was collected for the mechanical properties of geopolymer concrete and split into five groups. The dependent variables are splitting tensile strength, flexural strength, modulus of elasticity, Poisson’s ratio and strain corresponding to peak compressive stress, while the independent variable was kept to be compressive strength, but for the elastic modulus there is another independent variable which is concrete density.

Totally, 598 data observation for splitting tensile strength (from 54 studies), 285 for flexural strength (from 22 studies), 265 for elastic modulus (from 25 studies), 111 for elastic modulus-density (from eight studies), 99 for Poisson’s ratio (from nine studies), and 27 for strain corresponding to peak stress (from five studies) were collected from a total of 71 past studies for regression analysis. Details of test variables attempted by the investigators are given in Table 1, arranged according to the date of publication. Those data related to the specimens tested at the age of 3 days by some researchers have not been included in the database of this study. It is of interest to give a description of test variables shown in Table 1. Out of 71 studies, 30 used chemical admixture to enhance workability. Majority of researchers have used fly ash as a binder; however, 28 studies used GGBS alone or with fly ash. Moreover, there are studies worked on metakaolin, rice hush ash, natural Pozzolan, limestone powder, bagasse ash, POFA and nanomaterials. Na_2_SiO_3_/NaOH ratio varies between 0.15 to 3.33, while molarity varies from 3.2 to 20. The water/soild ratio was found to vary between 0.025 and 0.6, while the alkali solution/binder ratio varies from 0.085 to 0.944. Coarse aggregate maximum size varied from zero (on using cement mortar) to 25 mm, but majority of researchers have used 20 mm size aggregate. With regard curing of specimens, different curing regimes were followed, and in general, there is curing at ambient temperature and heat curing of maximum temperature equal to 100 °C and a duration of 72 h.

**Figure 1:**
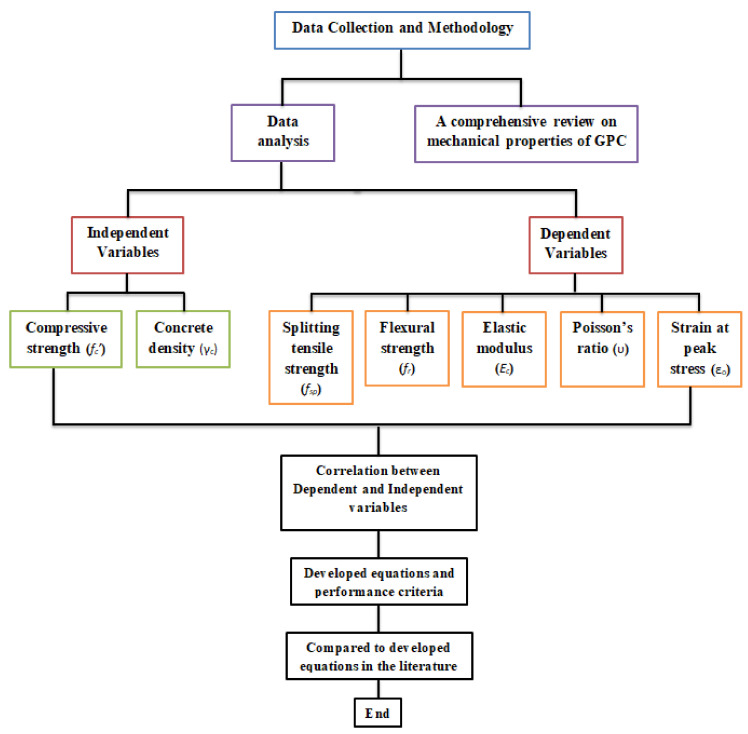
The flow chart diagram process followed in this study.

**Figure 2:**
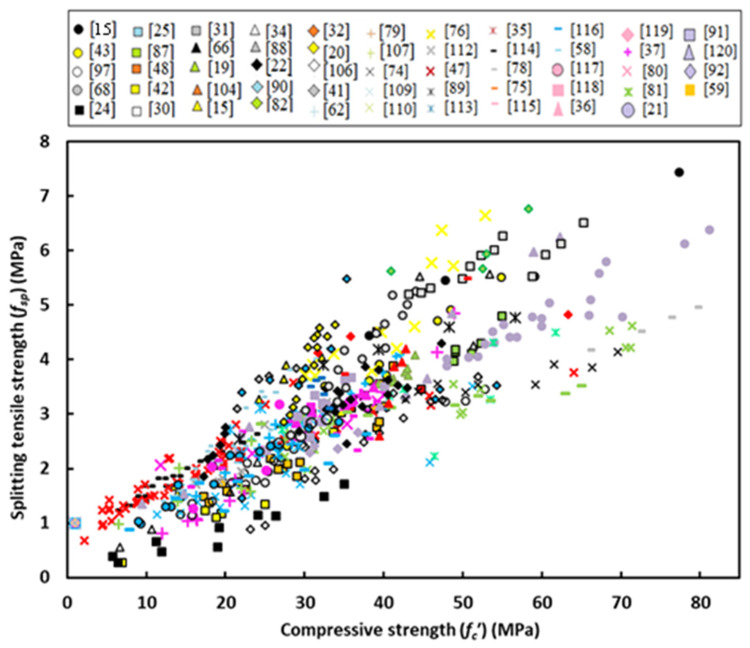
Variation of splitting tensile strength with compressive strength of GPC.

**Figure 3:**
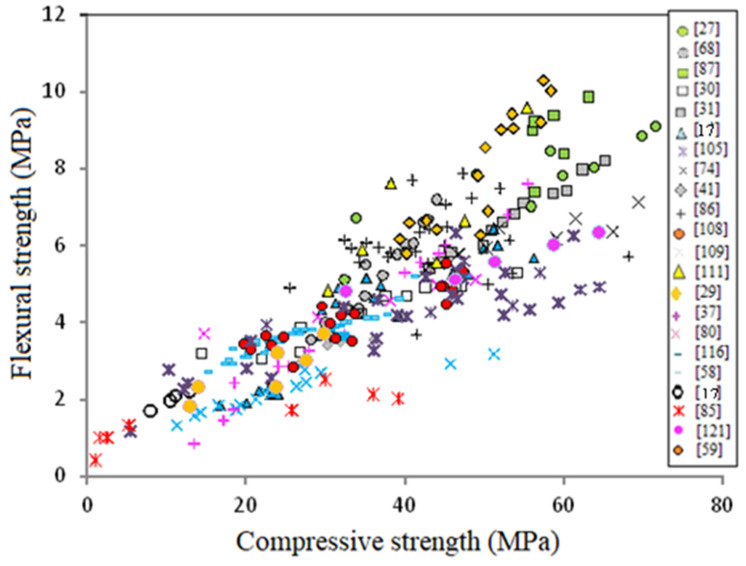
Variation of flexural strength with compressive strength of GPC.

**Figure 4:**
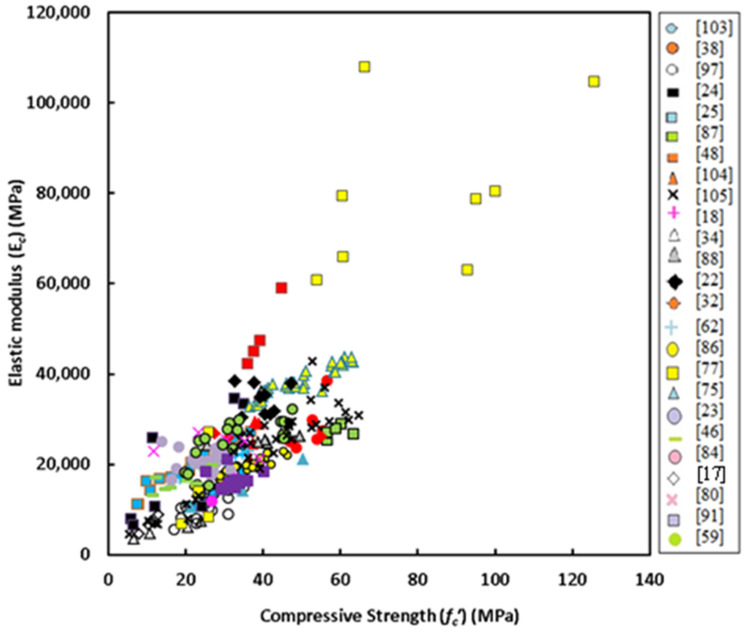
Variation of elastic modulus with compressive strength of GPC.

Further details of the data collection and modeling work are summarized in the form of a flow chart as depicted in Figure 1. It should be noted that the unique independent variable is the concrete compressive strength (*f_c_’*) but for the elastic modulus another equation is obtained from regression analysis depending on concrete density (γ_c_) beside the compressive strength. Figure 2, Figure 3 and Figure 4 show variation of splitting tensile strength, flexural strength and elastic modulus with compressive strength of GPC, respectively.

## 5. Regression Analysis and Proposed Equations

Different specimens with regard to the size and shape were used by the researchers (see Table 1), and the standard case was kept as the 150 mm × 300 mm cylinder. To make a conversion of the test data to obtain the standard for the properties of compression, splitting tensile strength, and elastic modulus, there was a need to use the related equations given by some researchers. For this purpose, conversion relationships described by Neville [122], Mohammed et al. [123], Hamad [124], and Graybeal and Davis [125] were used to convert test data to those of standard case. For the case of dog bone shape specimen used for direct tension by some researchers, the results were divided by 0.9 to obtain the splitting tensile strength recommended by Eurocode 2 [126], and then changed to the standard cylinder. Conversation was not made for those data of absent specimen’s properties.

**Figure 5:**
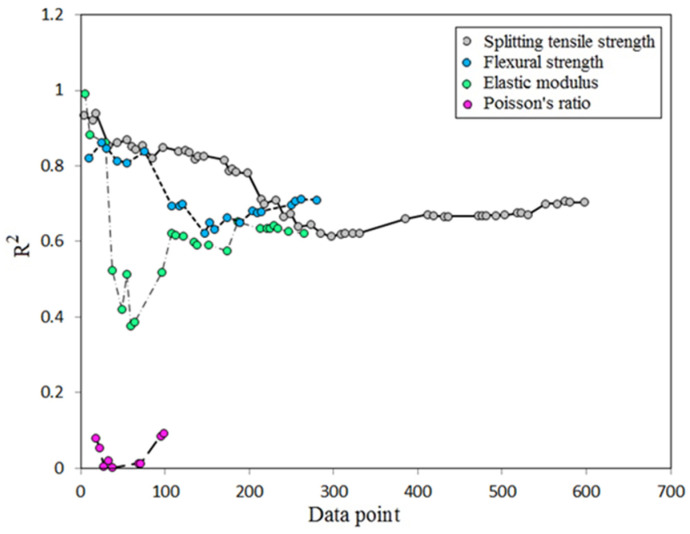
Variation of coefficient of determination for different properties.

It is of interest to show how the linear coefficient of determination (R^2^) varies with the number of data points. Those equations proposed based on the researcher’s own data are of high R^2^ [17,47,78], and when test data are mixed, there is a reduction of R^2^ as observed by Topark-Ngarm et al. [22] and Cui et al. [91]. Figure 5 shows variation of R^2^ with number of data observation collected for different mechanical properties. For all test data used for analysis, R^2^ was found to be 0.704, 0.71 and 0.622 for the splitting tensile strength, flexural strength, and elastic modulus respectively, while there is a very low value of R^2^ for the Poisson’s ratio which is only 0.093. The best choice of the two equations (linear and power) is that one gives highest R^2^, and on this base there is a chance for R^2^ to be higher than the mentioned linear R^2^. The two equations have the following form:

y = a + b x(1)

y = k (x)^n^ (2)

### 5.1. Splitting Tensile Strength

**Figure 6:**
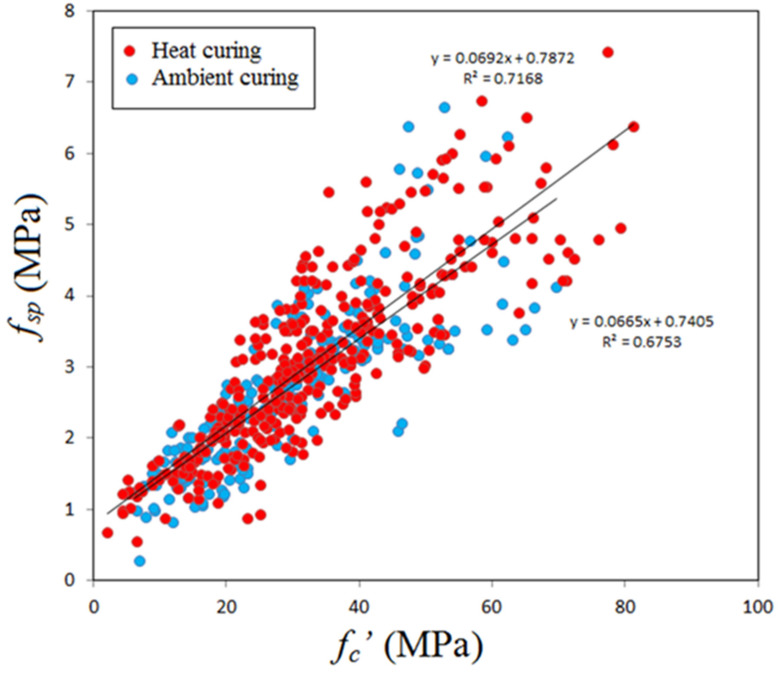
Variation of splitting tensile strength with compressive strength.

To be more precise, it is necessary to show data scatter of splitting tensile strength for GPC subjected to initial heat curing or ambient curing, because curing regime was found to have an important effect on each mechanical property as observed by many investigators. Figure 6 shows variation of splitting tensile strength with compressive strength for GPC subjected to heat curing or ambient curing. One can observe that the effect of curing is not important on the splitting tensile strength-compressive strength relationship and the two equations obtained from regression analysis are close to each other. Consequently, there is a chance to neglect the effect of curing and accordingly the following power equation was obtained for all data scatter

**Figure 7:**
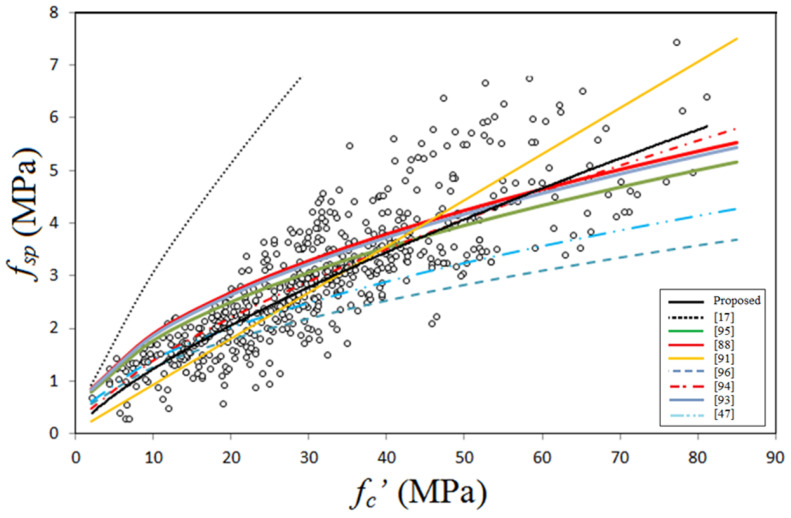
Splitting tensile strength data scatter and proposed equations.

*f_sp_* = 0.222 (*f_c_’*)^0.7436^ (3)

where *f_sp_* and *f_c_’* are measured in MPa. For this equation R^2^ was found to be 0.712, which is relatively large because regression analysis was made on 598 data points. Figure 7 shows variation of splitting tensile strength with compressive strength in addition to the proposed equation curve, together with the predictions of some proposed models given by the codes of practice and researchers. One can observe that the equation proposed by Jamal [17] is highly overestimates tests data and accordingly there is no safety to apply this equation for splitting tensile strength of GPC. The reason for this overestimation is that the proposal is based on based on a small amount of test data on lightweight GPC. In contrast, the proposals of AS3600 [96] and Jindal et al. [47] highly underestimate the test data accompanying high safety. One can find that the predictions of CEP-FIP [94] is quite close to the proposed equation (Equation (3)) and considered to be safe and accurate for predicting splitting tensile strength of GPC. The linear equation proposed by Cui et al. [91] is underestimates test data for concrete of compressive strength lower than about 35 MPa and moderately overestimates test data for larger strength concretes. One can observe that the equations given by ACI 363 [93] and Albitar et al. [88] are nearly the same, and they overestimate thes test data for GPC of compressive strength lower than about 50 MPa.

### 5.2. Flexural Strength

**Figure 8:**
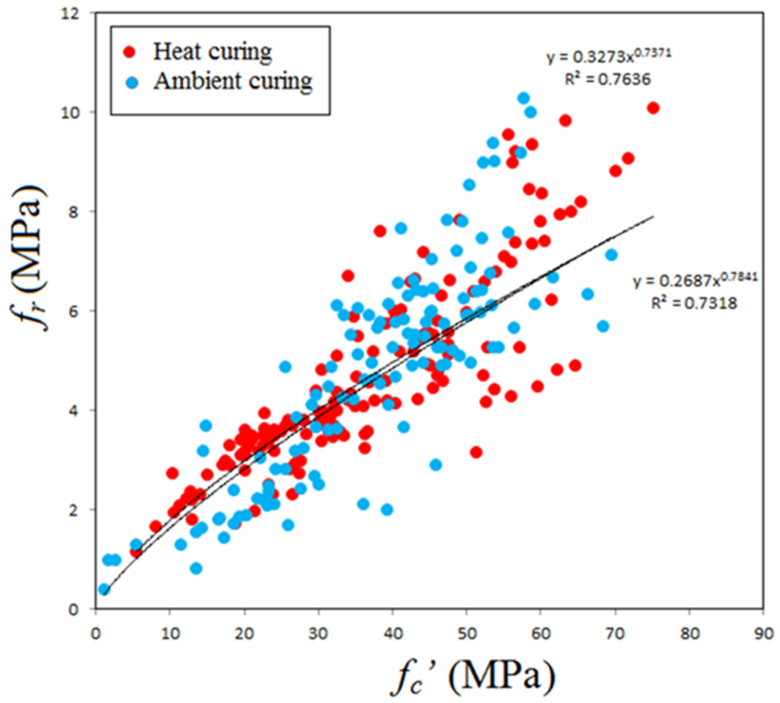
Variation of flexural strength with compressive strength.

Figure 8 shows variation of splitting tensile strength with compressive strength for GPC subjected to heat curing or ambient curing. One can observe that the effect of curing is not important on the flexural strength-compressive strength relationship and the equations obtained from regression analysis are close to each other, similar to the case of splitting tensile strength. Consequently, there is a chance to neglect the effect of curing, and on using, the following power equation was obtained

**Figure 9:**
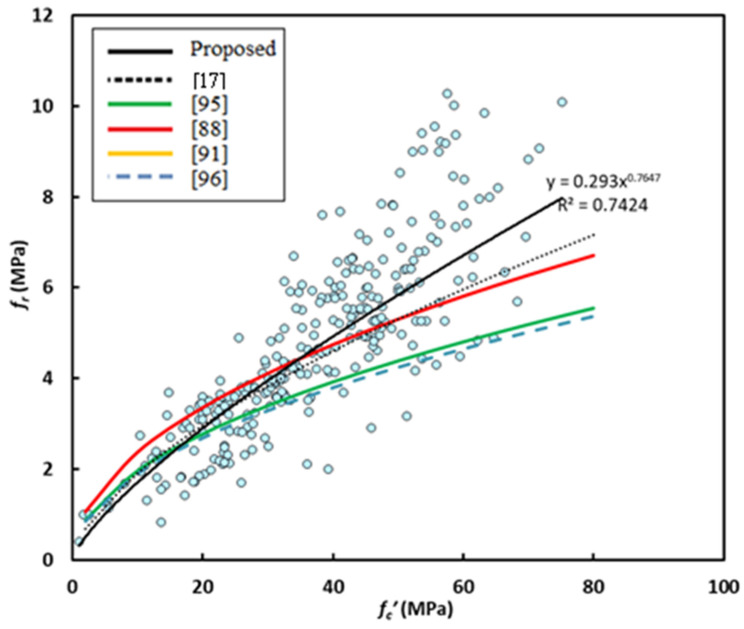
Flexural strength data scatter and proposed equations.

*f_r_* = 0.293 (*f_c_’*)^0.7647^ (4)

where *f_r_* and *f_c_’* are measured in MPa. For this equation R^2^ was found to be 0.742, which is relatively large because regression analysis was made on 280 data points. Figure 9 shows variation of flexural strength with compressive strength in addition to the prediction of some past equations given by researchers or codes of practice. The predictions of Albitar et al. [88]’s equation overestimate the test data for GPC of compressive strength up to about 35 MPa, while the other predictions give nearly the same flexural strength for concrete of compressive strength lower than about 20 MPa. The predictions of ACI 318 [95] and AS3600 [96] are close to each other and highly underestimates test data accompanying with safety. The equation of Jamal [17] is more accurate and closer to the proposed equation (Equation (4)) for GPC of compressive strength lowers than 35 MPa, and moderately underestimates the tensile behavior for larger strengths.

### 5.3. Elastic Modulus

**Figure 10:**
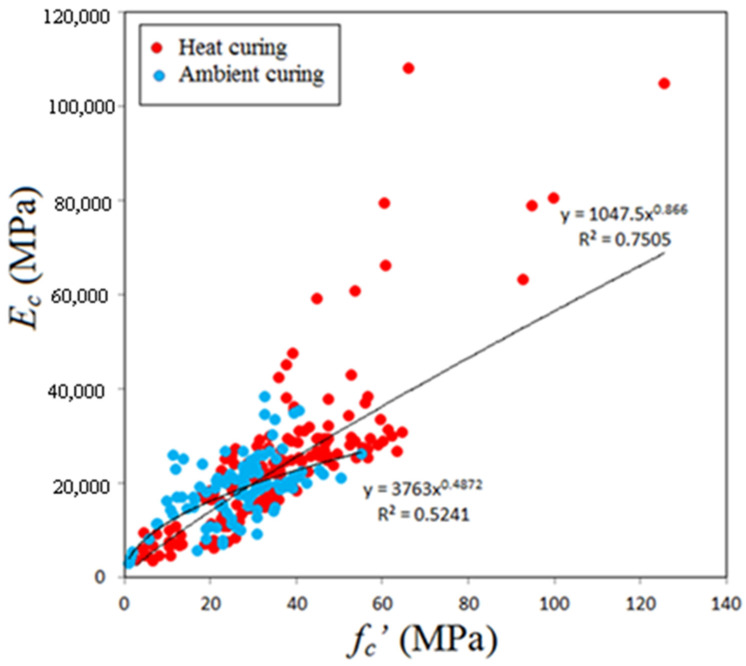
Variation of Elastic modulus with compressive strength.

Figure 10 shows variation of the elastic modulus with compressive strength for GPC subjected to heat curing or ambient curing. The data scatter is somewhat different from the previous two cases, because for the case of GPC subjected to ambient curing maximum compressive strength tested is close to 55 MPa, in addition, there is a relatively weak correlation between the elastic modulus and compressive strength. For concretes of compressive strength lower than 55 MPa, there is a mixed data scatter and the effect of initial curing regime seems to be not important, identical to the two previous cases. Based on this observation, there is a chance to make regression analysis on the whole test data and neglect the effect of curing. For this case, the linear equation given below was found accurate of highest R^2^ which is 0.642.

**Figure 11:**
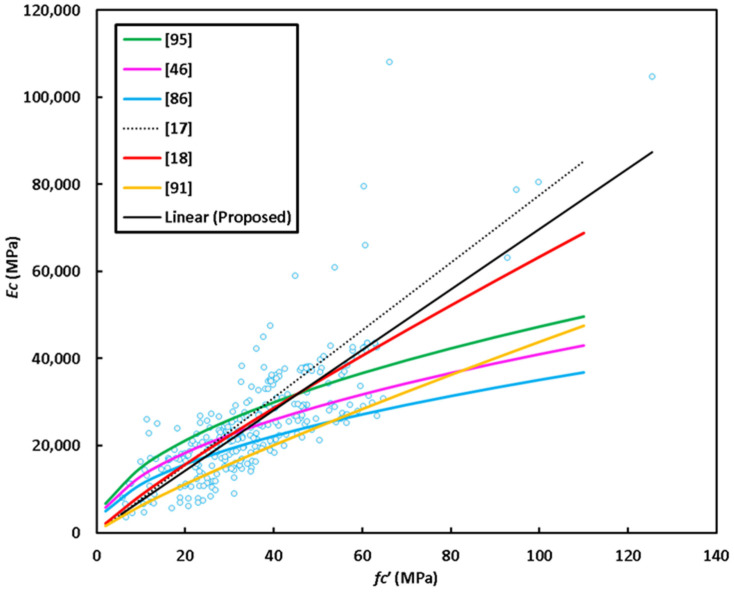
Elastic modulus data scatter and proposed equations.

*E_c_* = 479.4 + 692.41 *f_c_’* (5)

where *E_c_* and *f_c_’* are measured in MPa. Figure 11 shows variation of the elastic modulus with compressive strength in addition to the predictions of some past equations given by the researchers or codes of practice. The predictions of Posi et al. [18] is similar to that of proposed equation (Equation (5)), but slightly underestimates test data for GPC of compressive strength greater than about 50 MPa. ACI 318 [95] equation overestimates test data for concrete of compressive strength lower than 50 MPa, while the proposal of Cui et al. [91] highly underestimates tested elastic modulus. In general, the equation of Jamal [17] proposed basically for lightweight concrete moderately overestimates test data. The model given by Nath and Sarkar [86] and Hassan et al. [46] are moderately accurate but tend to underestimate test data for GPC of compressive strength higher than about 40 MPa.

**Figure 12:**
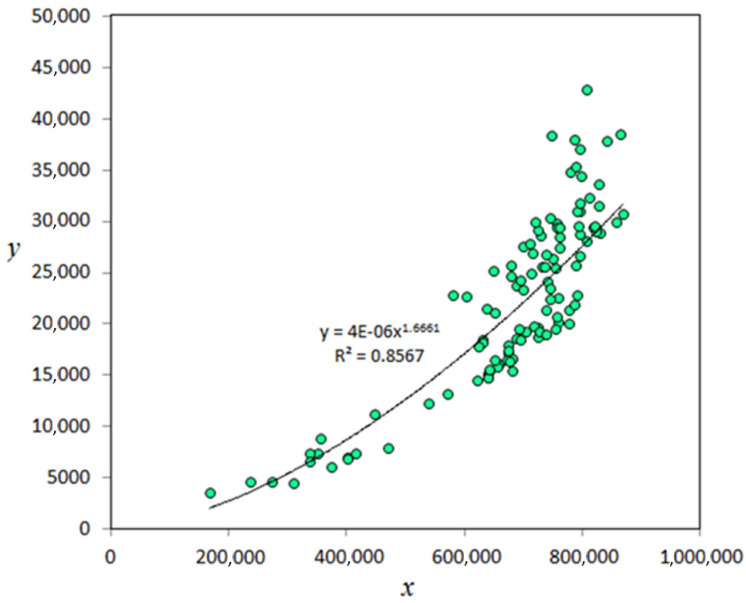
Elastic modulus- x relationship.

It is of interest to perform regression analysis to develop an equation for the elastic modulus taking into account the effect of concrete density, because there are proposed equations based on both compressive strength and density of concrete such as that given by ACI 318 [95]. Data collected for the elastic modulus has 99 data points, because some experiments contain no information about the density. The authors attempted to combine compressive strength (*f_c_’*) and density (*γ_c_*) in a single independent variable (x), and for this purpose many trials were made. For the best correlation between dependent variable (y) which is the elastic modulus and x, the later was found to be [(*γ_c_*)^1.6^(*f_c_’*)^0.3^]. Figure 12 shows data scatter for the y-x relationship. Regression analysis was carried out and the final form of the proposed equation was found to be

**Figure 13:**
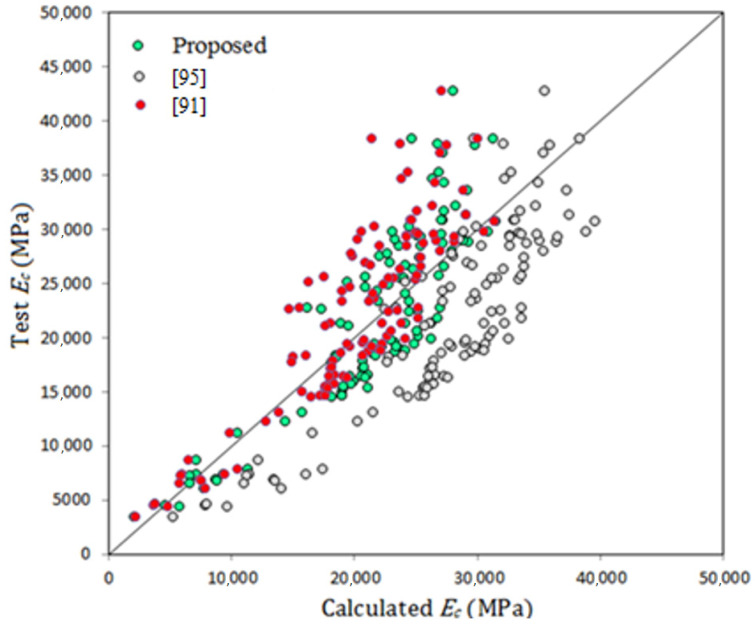
Test and calculated elastic modulus of GPC.

*E_c_* = 4 × 10^−6^ · (*γ_c_*)^2.666^ · (*f_c_’*)^0.5^ (6)

where *E_c_* is measured in MPa and R^2^ for this equation is 0.857 better than that of Equation (5), indicating a strong relation between elastic modulus and concrete density. It is worth checking the accuracy of some proposed equations when used for calculating elastic modulus of GPC. Figure 13 shows the test elastic modulus versus the calculated modulus from Equation (6), ACI 318 [95] and Cui et al. [91]. The predictions of ACI 318 [95] in contrast to those by Cui et al. [91] seem to be not safe. For the three predictions mean (test/calculated) elastic modulus is 1.03, 0.778 and 1.12 for the current proposal, ACI 318 [95] and Cui et al. [91], respectively, indicating the accuracy of the proposed equation and safety of that given by Cui et al. [91]. In contrast, there is no chance to apply safely the equation of ACI 318 code [95] for the elastic modulus of GPC when density of concrete is considered.

### 5.4. Poisson’s Ratio

**Figure 14:**
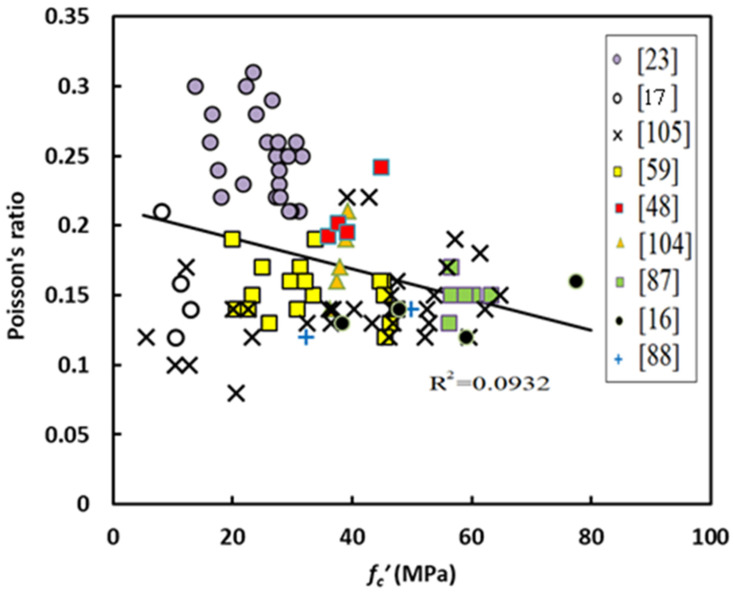
Variation of Poisson’s ratio with compressive strength of GPC.

**Figure 15:**
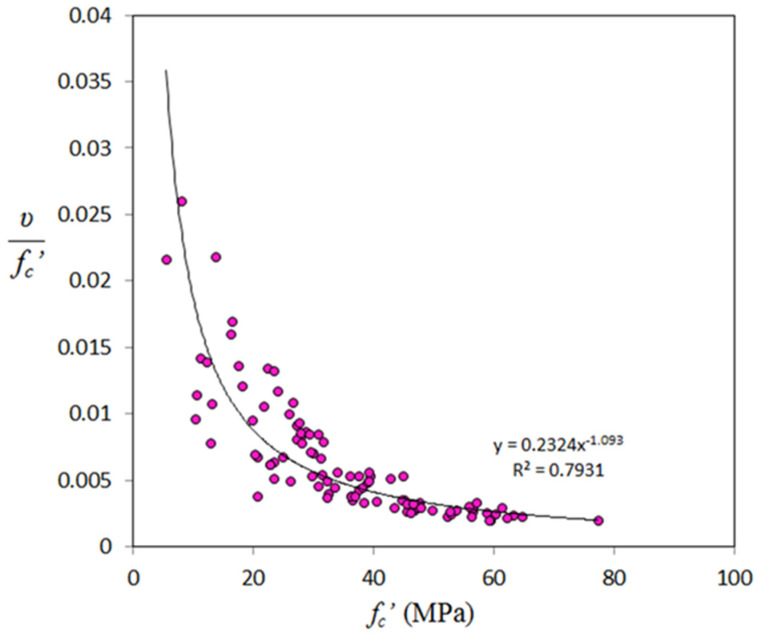
Variation of normalized Poisson’s ratio with compressive strength.

Compared with the other properties of geopolymer concrete, there is limited test data for this property. In this study, total of 99 data samples were collected. Additionally, it seems that equations for the prediction of Poisson’s ratio of GPC are not available. Figure 14 shows variation of Poisson’s ratio with compressive strength, from which one can observe, in contrast to the other mechanical properties, a very weak correlation. The authors think that there is no chance to carry out regression analysis on this sort of data. For this purpose, and to solve this shortcoming, it is better to construct a correlation between the normalized Poisson’s ratio (*υ/f_c_’*) and compressive strength. Figure 15 shows variation of normalized Poisson’s ratio and compressive strength, and one can find a high coefficient of determination (R^2^). From regression analysis, the final form of the equation for predicting Poisson’s ratio is given by

(7) $$\upsilon =\frac{0.2324}{{\left(f_{c}^{\prime }\right)}^{0.093}}$$

R^2^ for the above equation is 0.793, and *f_c_′* is measured in MPa. The mean (test/calculated) value of Poisson’s ratio for GPC was found to be 1.04, indicating the accuracy of the proposed equation accompanying with safety.

### 5.5. Strain Corresponding to Peak Compressive Stress (ε_o_)

**Figure 16:**
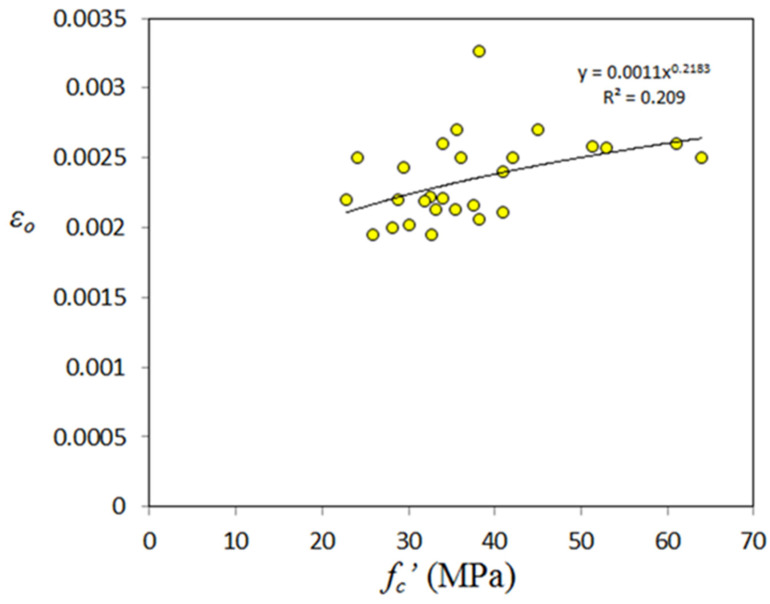
Variation of strain corresponding to peak stress with compressive strength of GPC.

This property has a great importance to assess accurately the compressive stress-strain relationship of GPC. Those models proposed for compressive stress-strain response of GPC are based on some equations proposed basically for normal concrete, and the parameter of strain corresponding to peak stress (ε_o_) taken is that of normal concrete. The authors think that it is better to derive equations for this parameter, since test data obtained by some researchers are available. Additionally, there is a chance to assess the accuracy of the assumed ε_o_ basically derived the for normal concrete. To do this, there is a need to review the related works performed on the compressive stress- strain relationship of GPC. This property was experimentally investigated by Hardjito [15], Yang et al. [25], Albitar et al. [88], Albitar et al. [32], and Parveen et al. [114]. From the stress-strain curves, strain corresponding to peak stress (ε_o_) was picked up and found that there are 27 strain values. Figure 16 shows variation of ε_o_ with compressive strength, from which one can find that there is a weak correlation, identical to that of Poisson’s ratio.

**Figure 17:**
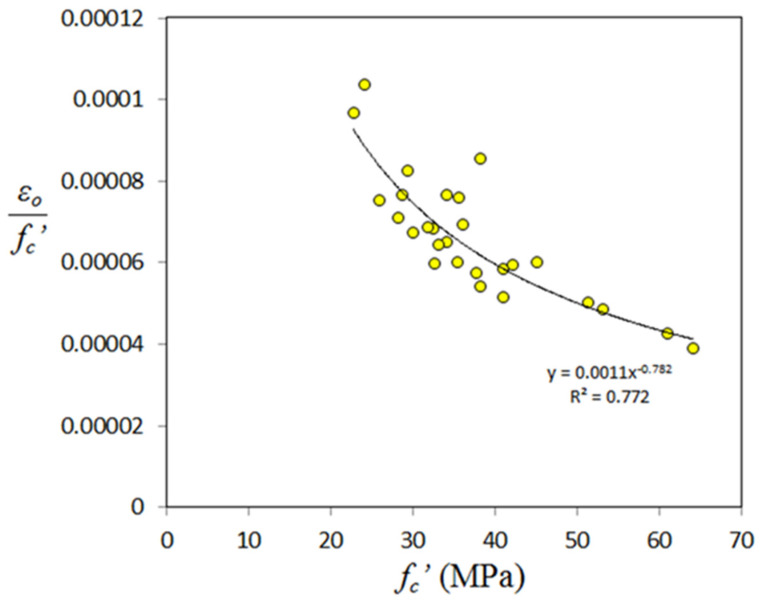
Variation of normalized *ε_o_* with compressive strength of GPC.

Similar to the case of Poisson’s ratio, it is necessary to construct the correlation between normalized strain corresponding to peak stress with compressive strength as shown in Figure 17, from which one can find a relatively high R^2^ value. Regression analysis was made and the final form of the proposed equation is as follows:

*ε*_o_ = 0.0011 (*f_c_’*)^0.218^ (8)

For the above, Equation R^2^ was found to be 0.772 and mean (test/calculated) value is 0.975. Now, it is worth checking the accuracy of some proposed equations for calculating εo applied on test data on GPC. Mean value of test/calculated strain was found to be 0.999 for the equation proposed by Albitar et al. [88], indicating that there is a good chance to apply their proposed equation accurately. Mean value of test/calculated strain was found to be 1.05 and 1.1 for the equation of CEB-FIP [94] and Eurocode 2 [126], respectively, indicating that these equations can be used safely for the strain corresponding to peak compressive stress.

## 6. Conclusions

Alkali-activated materials are considered to be very suitable from the point of view of sustainable development. This novel concrete meets many of the requirements including high strength, use of secondary materials, low carbon trace, minimum greenhouse gas emissions, good frost resistance, etc. A revised data analysis on the mechanical properties of geopolymer concrete was made in this study and equations were proposed for each property. The outcome of the study is interesting, because the proposed equations derived for the mechanical properties are accurate since they were based on a relatively large amount of test data. These equations can be used in the design of GPC structural members and to prepare a state-of-art report on GPC. Furthermore, the following conclusions can be drawn.

I. Different properties of geopolymer concrete (GPC) can be correlated with compressive strength; however the correlation strength usually reduced as the number data taken for analysis increased.
II. The effect of initial curing temperature was not important for correlation between different properties and compressive strength, and from this basis, a single equation was proposed using regression analysis. For the splitting tensile strength and flexural strength, power equation (Equations (3) and (4)) was found accurate, while there is a linear equation (Equation (5)) for the elastic modulus.
III. There is a better prediction of the elastic modulus based on both compressive strength and concrete density. Because of very low correlation, it was better to correlate normalized Poisson’s ratio and strain corresponding to peak stress with compressive strength. From this basis, Equations (7) and (8) were obtained from regression analysis.
IV. Accuracy of most important past equations were checked against the large number of test data collected, and found that the equation of CEB-FIP [94] is accurate for predicting splitting tensile strength, while the proposal of ACI 363 [93] overestimates test data for compressive strength smaller than 50 MPa, at the time, equation of AS3600 [96] is highly underestimates test data.
V. In general, most of the proposed equations are safe for predicting flexural strength, while the equation given by ACI 318 [95] overestimates test data for concrete of compressive strength up to about 40 MPa. Again, there is no chance to apply this equation for elastic modulus if density is considered. Furthermore, there is a good chance to apply some equations proposed for the strain corresponding to peak stress proposed for normal concrete safely.

## Figures and Tables

**Table 1:** Details of test variables.

Reference	Chemical Admixture	Binder	Na_2_SiO_3_/ NaOH	Molarity	Water/ Solid	Alkali Solution/ Binder	MS (mm)	Initial Curing	Specimen (*f_c_’, f_sp_, f_r_, E_c_*)
[15]	8.2/408 and 6.1/408	Class F Fly ash	103/41 and 103/55.4	8,14	--	41/408 and 55.4/408	10	60 °C (Oven) for 24 h 90 °C (Steam) for 24 h	A, B,-,-
[103]	-	Class F fly ash	0, 0.15	8, 12.5	0.55	0.4	12	85 °C (Oven) for 20 h	I,-,W,B
[27]	NA	Slag and metakaolin	-	3.2	0.35	-	NA	20 °C and RH 100% for 28 days 80 °C and RH 100% for 2, 4, 8 h. Autoclave curing at 150 °C for 2 h	C,-,D,-
[38]	-	Class F fly ash	-	-	Variable	Variable	14	30 °C–35 °C (Steam) for 24 h	-,B,T,-
[43]	6 kg/m^3^	Class F Fly	102/41	14	-	0.35	20	80 °C (Steam) for 24 h without or with rest for 1 day.	A,B,-,-
[97]	-	Fly ash, Rice husk–bark ash	2.5	14	-	0.18 to 0.212	-	Ambient curing	-
[68]	-	Class F fly ash	2.5	8 to 16	-	0.35	20	25 °C and 60 °C (Oven) for 24 h	I,-,-,-
[24]	-	Natural pozzolan	0.476 to 0.516	-	0.42 to 0.55	Variable	14	Sealed curing (20 °C, 60 °C)	G,A,-,A
[25]	0.3% to 2%	GGBS	1/7.5	-	Variable 0.25 to 0.6	8.5% and 2%	25	Ambient curing	A,A,U,-
[87]	6.1/498.46 to 6.1/394.29	Class F Fly ash with OPC	1.5–2.5	14	15.97/ 426.62 to 28.51/ 428.57	0.3–0.4	20	60 °C for 24 h. 70 °C for 12 h. 75 °C for 24 h.	A,B,F,A
[48]	2%	Low calcium fly ash	2.5	10	0.25	0.55	20	100 °C	I,B,H,-
[42]	3%	Class F fly ash	2.5	12	0.1	0.4	19	Ambient curing and 60 °C for 24 h	I,B,H,-
[30]	6/360	Class F fly ash& GGBS	2, 2.5	14	0.197, 0.202	0.37,0.4	20	Ambient curing at 17–22 °C and 70 ± 10% RH	-
[31]	-	Class F fly ash and GGBS	0.15	12.2	-	0.5	14	80 °C (Sealing) for 48 h.	-.-.H.-
[66]	NA	Class F fly ash	1	9	-	0.56	19	Air curing for 24 h followed by 60 °C steam for 48 h.	A,A.-,-
[13]	-	Class C Fly ash with Nanosilica or Nanoalomina	2	10	-	0.6	-	Normal curing at 27 °C	L,-,M,D
[19]	6%	Fly ash and silica fume	2.5	-	0.12	57/400	14	70 °C (Oven) for 48 h	G,A,H,-
[104]	7.67/639	Class F fly ash	2.5	14	0.39	252/639	20	Heat curing at 60 °C for 24 h	I,B,H,B
[105]	15/494	Class F and class C fly ashes	1	14	-	198/494 -464/494	10	60 °C for 72 h	B,B,F,B
[18]	-	Class F fly ash	0.33 to 3	5,10,15	-	2 to 2.8	12.5	Oven curing (25, 40, 60 °C) for 48 h	G,-,-B
[14]	-	Class F Fly ash& Nanosilica	1.75	8,10,12	-	0.4	-	Ambient curing at 27 °C	J,-,K,-
[34]	1.5%	Class-F FA and POFA	2.5	14	0.1	0.55	9.5	65 °C for 48 h	G,A,H,B
[88]	0 to 0.115	Low calcium fly ash	1.5	14	0 to 0.14	0.37	-	Ambient curing and 70° for 24 h	A,A,-,A
[22]	-	Class C fly ash	1,2	10,15,20	-	107/414, 208/414	20	60 °C (Oven) or ambient curing for 24 h	A,A,-,B
[90]	-	Low calcium fly ash	2	14	-	0.3,0.35,0.4	-	30°C and 60 °C (Oven) for 24 h	J,-,H,-
[82]	1%	Class F Fly ash & Silica fume	0	14	0.2	0.4	20	100 °C (Oven) for 72 h	G,B,H,-
[32]	-	Low calcium fly ash and granulated lead smelter slag (GLSS)	1.5	14	-	0.37,0.5,0.75	-	Ambient curing and 70 °C for 24 h and 48 h	-
[20]	6%	Fly ash with silica fume or with GGBS	2.5	12	0.12	200/450	12.5	Heat curing for 48 h	I,B,H,-
[106]	2.5%	Class F fly ash, bagasse ash, rice husk ash	2.5	12	-	0.3	20	60 °C (Oven) for 24 h	I,B,H,-
[41]	-	Class C Fly ash with OPC and inorganic aluminasilicate polymer	Variable	10	Variable	Variable	15	100 °C (Oven) for 24 h & 40 °C water for 28 days	G,A,H,-
[86]	Variable upto 6%	Class F Fly ash, OPC, GGBS	2.5	14	Variable upto 0.55	140/400 & 160/400	10	Ambient curing at 18–23 °C and 70 ± 10%RH	A,A,F,-
[62]	2.5/400 to 47/400	Class F fly ash	2.48 to 3.31	12,16,18	0.025 to 0.0875	0.3 to 0.45	9.5	Heat curing at 50 °C for 48 h	G,N,-,A
[79]	-	Class F fly ash and GGBS	2.5	10	55/409	0.35	20	Ambient curing	-
[107]	-	Fly ash and GGBS	2.5	-	0.25	-	-	Ambient curing	L,P,Q,-
[108]	-	Granite and slag powder	-	-	-	-	-	Heat curing at 60 and 80 °C for 8 h	D,-D,-
[74]	-	Class F fly ash and GGBS	2.5	10	55/409	0.35	20	Ambient curing	I,B,H,-
[109]	-	Class F Fly ash& GGBS	2259.2/320	8	-	-	20	Indoor, outdoor and oven	B,B,E,-
[110]	0,1.47%,1.52%	Fly ash	2, 2.5	8,16	0.158 to 0.31	0.35,0.4,0.45	-	60 °C (Oven) for 24 h	-
[111]	-	Class F fly ash with GGBS	2.5	12	-	0.45	20	Heat curing for 48 h	I,B,H,-
[76]	-	Metakaolin and GGBS	2.5	10	-	186/414	20	Ambient curing	I,B,H,-
[112]	7.9/197.5	Class F Fly ash & GGBS	129/52	8,4	-	52/197.5	20	Ambient curing at 30 °C and RH of 65%	I,B,-,-
[47]	-	Class F Fly ash	Variable	16	27.07/400 to 36.02/350	Variable	14	90 °C (Oven) for 24 h	I,-,H,-
[89]	-	Class F fly ash and GGBS	171/69	-	-	240/480	20	Ambient curing	I,B,H,B
[113]	-	Fly ash	2.5	-	-	0.6	20	heat curing at 80 °C for 24 h	I,-,-,-
[35]	-	WBG, Class F fly ash, GBFS, waste ceramic	3	Variable 2 to 16	-	0.55	-	Ambient curing	L,N,D,-
[114]	2%	Class F Fly ash	2.5	8,12,16	Variable	0.45	14	Ambient curing at 27 °C	I,I,-,-
[77]	3.04/380.66	Class F fly ash and GGBS	-	10	-	219.31/380.66	20	60 °C (Oven) for 24 h	G,A,-,B
[78]	6/500 and 9/500	Class F fly ash and GGBS	1.5 to 2.5	14	-	0.45	10	70 °C (Oven) for 48 h	G,A,H,-
[75]	0.83% to 0.96%	Class F fly ash and GGBS	2.5	8	-	0.55 to 0.944	-	Ambient curing	I,B,H,B
[115]	2%	Class F fly ash	2.5	16	0.05	0.45	20	60 °C (Oven) for 24 h	I,B,H,-
[29]	-	Class F Fly ash, Palm kernel shell ash, Rice husk ash	160.61/ 53.54	8,12	-	214.15/ 428.31	-	Ambient curing	-
[23]	1%	Class C fly ash	1	10,15	-	0.45–0.6	20	Ambient curing	A,-,-,A
[46]	-	Class F fly ash	117.4/67.1	10	79.2/410	0.4	16	75 °C (Oven) for 26 h or ambient curing.	I,-,H,A
[116]	0.5%	Class F fly ash	2	16	0.1	0.5	10,20	100 °C (Oven) for 24 h	I,B,O,-
[58]	SCC (3%)	Class F Fly ash, MK, GGBS	2.5	8,10,12	1% to 12%	0.47	-	70 °C (Oven) for 24 h	G,B,H,-
[84]	-	Class F fly ash with calcium aluminate cement	124.55/ 44.51	-	132.43/ 563.54	169/563.54	-	75 °C (Curing box) for 16 h	G,I,-,V
[117]	-	Class F fly ash and GBBS	2.5	12	-	0.46	20	60 °C (Oven) for 24 h	I,B,H,-
[118]	-	Class F fly ash, metakaolin	-	14	-	-	20	80 °C for 24 h	I,B,O,-
[36]	-	GGBS, SCBA	2.5	5	75/433.5	238.44/ 433.5	20	Sunlight curing	I,B,H,-
[119]	-	High calcium fly ash	-	-	0.25	-	-	Ambient curing	L,-,-,-
[37]	-	GGBS, Bagasse ash	170.32/ 68.12	5,10,15	75/ 433.54	68.12/ 433.54	20	In direct sunlight	I,B,H,-
[17]	From 1% to 4%	Fly ash, Limestone powder	1	8–16	0.167 to 0.23	0.35	12.5	80 °C (Oven) for 48 h	B,B,R,B
[80]	-	Class F Fly ash, GGBS	112.65/ 45.06	10	59.142/394.3	45.06/394.3	12.5	Ambient curing	G,A,H,B
[85]	-	Class F fly ash and GBBS	Variable	-	0.3	-	-	Ambient curing	C,-,D,-
[81]	-	Fly ash and GGBS	2.5	12	-	-	-	-	-
[21]	1.2%	GGBS with nano silica	2.5	10,12,16	0.27	0.45	14	60 °C Oven curing	-,B,H,-
[91]	-	Class F fly ash	2.5	12	31/420	210/420	14	80 °C (Oven) for 24 h	A,A,-,A
[120]	-	Metakaolin and Bottom ash	-	8	-	0.5	-	Ambient curing	-
[92]	-	Class C fly ash	1	10	78.3/450	0.3	20	70 °C (Oven) for 24 h	A,A,S,-
[121]	-	Class F fly ash	2	14	-	0.5	14	23 ± 2℃ and relative humidity 80 ± 5%.	B,B,R,-
[59]	2%	Class F fly ash	2.5	8,12,16	0.25	Variable	9.5	70 °C (Oven) for 24 h	B,B,E,-

POFA = palm oil fuel ash, WBG = Waste bottle glass, SCBA = Sugarcane Bagasse ash, A = 100 mm × 200 mm cylinder, B = 150 mm × 300 mm cylinder, C = 40 mm cube, D = 40 mm × 40 mm × 160 mm prism, E = 75 mm × 75 mm × 400 mm prism, F = 100 mm × 100 mm × 400 mm prism, G = 100 mm cube, H = 100 mm × 100 mm × 500 mm prism, I = 150 mm cube, J = 70.6 mm cube, K = 50 mm × 50 mm × 200 mm prism, L = 50 mm cube, M = 25mm × 50mm cylinder, N = 75 mm × 150 mm cylinder, O = 150 mm × 150 mm × 700 mm prism, P = 25 mm × 25 mm × 76 mm dog bone shape, Q = 75 mm × 75 mm × 285 mm prism, R = 150 mm × 150 mm × 500 mm prism, S = 150 mm × 150 mm × 600 mm prism, T = 100 mm × 100 mm × 300 mm prism, U = 150 mm × 150 mm × 550 mm prism, V= 150 mm × 150 mm × 300 mm prism, W = 150 mm × 100 mm × 700 mm prism.
